# Epidemiology and burden of influenza in the U.S. Department of Veterans Affairs

**DOI:** 10.1111/irv.12512

**Published:** 2017-12-05

**Authors:** Cynthia Lucero‐Obusan, Patricia L. Schirmer, Aaron Wendelboe, Gina Oda, Mark Holodniy

**Affiliations:** ^1^ Department of Veterans Affairs Office of Quality, Safety and Value Public Health Surveillance and Research Palo Alto CA USA; ^2^ Department of Biostatistics and Epidemiology University of Oklahoma Health Sciences Oklahoma City OK USA; ^3^ Division of Infectious Diseases & Geographic Medicine Stanford University Stanford CA USA

**Keywords:** burden of illness, epidemiology, influenza, public health surveillance, veterans

## Abstract

We describe influenza activity in the US Veterans Affairs (VA) population for the 2010‐2011 through 2015‐2016 seasons and compare with national CDC FluView data. VA confirmed influenza cases ranged from 1005 to 11 506 per season; triage calls from 6090 to 10 346; outpatient visits from 3849 to 13 406; antiviral prescriptions from 3650 to 32 826; hospitalizations from 546 to 4673; and deaths in hospitalized patients from 17 to 139. Peak activity was generally the same as observed nationally by the CDC. For the seasons analyzed, correlation between VA and CDC %ILI visits (*r* = .863), influenza hospitalizations (*r* = .953), positive tests (*r* = .948), and percent of tests positive (*r* = .938) was strong. Understanding influenza burden is important for evaluating prevention priorities and resource allocation within VA.

## 1 INTRODUCTION

Influenza is associated with considerable morbidity and mortality and remains important for public health surveillance and prevention efforts. Our experience with pandemic H1N1 influenza highlighted the importance of robust surveillance for informed decision‐making.^1^ Even in non‐pandemic years, influenza's clinical impact remains significant. A recent report estimated that from 2010 to 2016, annual U.S. influenza‐related illnesses ranged from 9.2 to 35.6 million, medical visits from 4.3 to 16.7 million, hospitalizations from 140 000 to 710 000, and pneumonia and influenza deaths from 4000 to 20 000.^2^ Although U.S. population‐based surveillance data have been used to estimate overall and age‐based influenza disease burden,^2^, ^3^, ^4^ the epidemiology and impact of seasonal influenza in more specific U.S. patient populations is less well described.

The U.S. Department of Veterans Affairs (VA) healthcare system serves 9 million enrollees at over 1200 Veterans Health Administration sites of care throughout the U.S. and U.S. territories.^5^, ^6^ Approximately 46% of Veterans are age 65 or older and 10% are female.^5^ VA patients may be at higher risk for influenza and influenza complications, because Veterans are older and have a higher overall disease burden than the general U.S. population.^7^ Since 2008, we have analyzed data for influenza activity and vaccination administration and produced routine surveillance reports. Surveillance activities during 2008‐2010 focused on outpatient and emergency department (ED) visits and syndromic surveillance for influenza‐like‐illness (ILI) which was utilized for situational awareness during the H1N1 influenza pandemic.^8^ In subsequent years, surveillance expanded to include inpatient, laboratory, antiviral, and telephone triage data.^9^ Data elements were integrated into a single surveillance application utilized for routine surveillance and evaluating trends, including in combined VA and Department of Defense patient populations.^9^, ^10^

The primary study objectives were to describe influenza burden in VA covering six influenza seasons (2010‐2016), and to evaluate validity of VA influenza activity indicators by comparing to national Centers for Disease Control and Prevention (CDC) FluView data for the same time period.

## 2 METHODS

We analyzed all influenza‐specific *International Classification of Diseases, Clinical Modification, 9th* and *10th Revisions* (ICD‐9‐CM codes 487‐488 and ICD‐10‐CM codes J09‐J11) outpatient and ED visits, hospitalizations (including intensive care unit stays and deaths), telephone triage encounters, ILI‐outpatient visits, influenza laboratory tests, and antiviral prescriptions covering the 2010‐2011 through 2015‐2016 seasons (starting epidemiologic week 40 each year) for all VA care sites using VA data sources (Praedico^™^ Surveillance System and Corporate Data Warehouse). These sources were previously evaluated and are routinely used for influenza surveillance.^8^, ^9^ Visits are grouped into the ILI syndrome if an assigned diagnosis code(s) matches a designated ILI syndrome code (Table S1).

Influenza vaccinations were obtained starting August 1 each year using Bar Code Medication Administration (BCMA) data and Current Procedural Terminology (CPT) codes. High‐dose vaccine formulation was captured based on drug name for inpatient BCMA data and assignment of the high‐dose formulation code (CPT 90662) for outpatients.

For national comparison, we used CDC FluView influenza hospitalization (FluSurv‐NET), ILI visit (ILINet), laboratory data (U.S. WHO/NREVSS Collaborating Laboratories), and surveillance summaries for the same seasons.^11^, ^12^ We calculated Pearson correlation coefficients and *P*‐values to describe correlation between VA and CDC influenza hospitalizations, laboratory testing, and percentage of outpatient visits for ILI (%ILI). Observations for each measure were weekly metrics. For %ILI correlation calculations, VA data were limited to visits in primary and urgent care settings to match provider types represented in ILINet. We calculated peaks for influenza activity during each season (highest weekly value reported by season), for each data source and compared timing of peaks across VA influenza indicators and between VA and CDC FluView data.

## 3 RESULTS

Influenza laboratory, hospitalization, outpatient, telephone triage, antiviral, and vaccination surveillance metrics for each season are presented in Table 1. Influenza A was the predominant influenza type identified in 25 786 (77%) of 33 397 laboratory‐confirmed cases. For 13 690 influenza‐coded hospitalizations, median age was 67 years, 12 914 (94%) were male, and median stay was 4 days, with 2148 (16%) including time in intensive care and 406 deaths (3%). There were 2 716 382 ILI and 52 634 influenza‐coded outpatient visits (including 25 711 ED visits). Less than one‐third of VA users (enrollees that sought and received VA care during each fiscal year) had a documented VA‐administered influenza vaccine each season. High‐dose vaccine formulation accounted for 4% of total vaccine given (Table 1).

**Table 1:** Veteran Affairs (VA) influenza surveillance metrics, 2010‐2011 through 2015‐2016 seasons

	2010‐11N (%)	2011‐12N (%)	2012‐13N (%)	2013‐14N (%)	2014‐15N (%)	2015‐16N (%)
Influenza tests performed^a^	18 529	16 077	38 876	44 746	70 836	62 058
Total positive	2041 (11)	1005 (6)	6361 (16)	6095 (14)	11 506 (16)	6389 (10)
A	1618 (79)	856 (85)	4841 (76)	4985 (82)	9058 (79)	4428 (69)
B	403 (20)	132 (13)	1448 (23)	1060 (17)	2355 (20)	1888 (30)
Both A and B or Not specified	20 (1)	17 (2)	72 (1)	50 (1)	93 (1)	73 (1)
Influenza‐coded hospitalizations^b^	853	546	2509	2442	4673	2667
Unique patients	841	538	2475	2404	4589	2614
Intensive care unit stay	146 (17)	99 (18)	403 (16)	491 (20)	629 (13)	380 (14)
Deaths	26 (3)	17 (3)	72 (3)	73 (3)	139 (3)	79 (3)
Median length of stay (d)	4	3	4	4	4	3
Median age (y)	64	64	67	64	69	66
Hospitalization rate per 100 000 VA users^c^	14.7	9.2	41.7	39.5	74.2	41.8
ILI‐outpatient visits (All locations)^d^	480 095	459 986	487 609	419 313	468 571	400 808
Primary care and urgent care	299 902 (62)	280 161 (61)	287 161 (59)	243 139 (58)	269 026 (57)	225 890 (56)
Influenza‐coded outpatient visits^e^	6465	3849	13 406	7129	12 101	9684
Unique patients	5644	3398	11 438	5703	9899	7921
Emergency department (ED)	2479 (38)	1449 (38)	6082 (45)	3942 (55)	6974 (58)	4785 (49)
Influenza telephone triage calls^f^	6090	7485	9455	8388	10 346	8 418
Unique patients	5914	7252	9158	8139	10 042	8236
Antiviral prescriptions^g^	7628	3650	21 117	16 753	32 826	16 983
Outpatient	5112 (67)	2750 (75)	14 910 (71)	12 418 (74)	25 287 (77)	12 910 (76)
Inpatient	2516 (33)	900 (25)	6207 (29)	4335 (26)	7539 (23)	4073 (24)
Influenza vaccinations^c^	1 574 759 (27)	1 886 553 (32)	1 935 957 (32)	1 964 796 (32)	1 820 470 (29)	1 774 795 (28)
High‐dose formulation	33 644 (2)	43 729 (2)	39 474 (2)	47 234 (2)	101 432 (6)	176 932 (10)
Outpatient	1 543 410	1 855 279	1 904 663	1 936 224	1 794 119	1 746 536
Inpatient	31 349	31 274	31 294	28 572	26 351	28 259

a Invalid, rejected, canceled, not performed, dummy/test patient tests and influenza antibody titers were excluded. Duplicate positives within 30 d of the original positive were excluded unless a different influenza type or subtype was identified.

b Includes inpatient stays with an ICD‐9‐CM or ICD‐10‐CM admitting or any discharge diagnosis code of influenza. Data include some non‐VA hospitalizations if electronic data were transmitted to VA. Intensive care includes bed section designations of medical intensive care unit (ICU) and step‐down unit, surgical ICU and step‐down unit, and/or cardiac ICU and step‐down unit. Deaths include those hospitalizations for which the discharge disposition was death or for whom the recorded date of death matched the date of discharge.

c Hospitalization rate and vaccination percentage is based on the reported end of year (EOY) VA users for each fiscal year [VHA Support Service Center (VSSC) Current Enrollment Cube]. Users are enrollees who sought and received care anywhere in the VA healthcare system during a given fiscal year. Duplicate vaccinations recorded on the same date were excluded. High‐dose formulation was based on the drug name for inpatient vaccines and an assigned CPT code of 90662 (Influenza virus vaccine, split virus, preservative free, enhanced immunogenicity via increased antigen content, for intramuscular use) for outpatient vaccines.

d Includes visits in any VA outpatient setting which were assigned ICD‐9‐CM or ICD‐10‐CM code(s) that matches a designated ILI syndrome code (Table S1). Primary and Urgent Care includes ILI visits limited to the following VA clinic designations: general internal medicine, primary care clinic, primary care group, women's clinic, geriatric primary care, geriatric problem‐focused clinic, urgent care unit, home‐based primary care, pediatrics, and family practice clinic.

e Includes encounters in any outpatient setting with an ICD‐9‐CM or ICD‐10‐CM diagnosis code of influenza. ED visits based on encounter location designation of Emergency Room.

f Includes telephone triage encounters assigned a diagnosis code of influenza. Telephone triage was not available in all VA regions during the 2010‐2011 and 2011‐2012 seasons.

g Outpatient antiviral prescriptions included oseltamivir or zanamivir. Duplicate prescriptions within the same week were excluded. Inpatient antivirals represent Bar Code Medication Administration (BCMA) data for oseltamivir, zanamivir or peramivir. Subsequent doses for the same patient within a 30‐d period were excluded, as were doses that were held, refused or not given.

**Figure 1:**
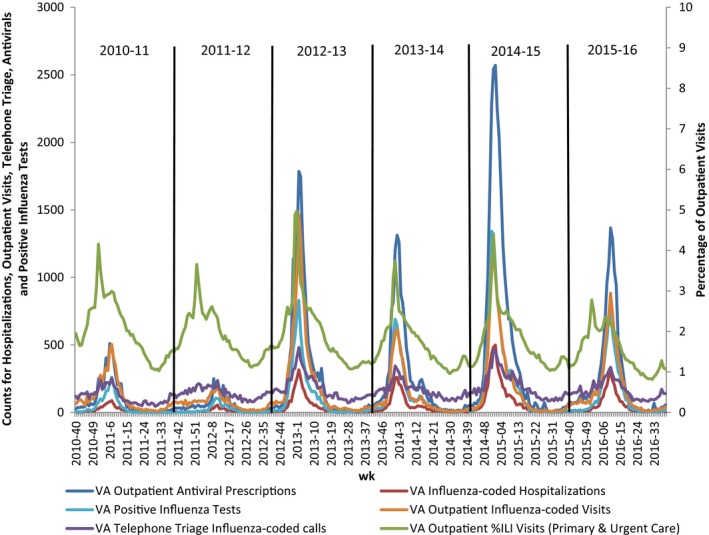
Comparison of key VA influenza indicators, 2010‐2011 through 2015‐2016 seasons

**Figure 2:**
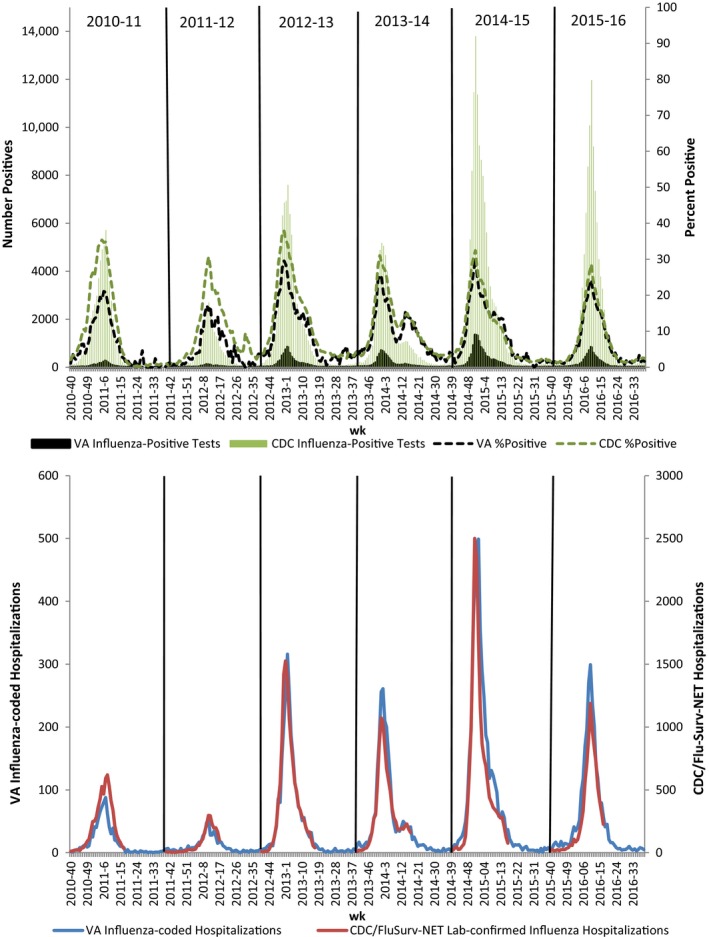
Comparison of select VA and CDC influenza indicators. Positive laboratory test and %positive (top) and influenza hospitalizations (bottom), 2010‐2011 through 2015‐2016 seasons

The majority of VA influenza metrics peaked the same week or within 2 weeks of each other for each influenza season, the exception being %ILI which peaked 6‐10 weeks earlier than other metrics during the 2010‐2011, 2011‐2012, and 2015‐2016 seasons. Peak week for influenza activity was late December to early January for 2012‐2013, 2013‐2014, and 2014‐2015 seasons. Activity peaked in early‐mid February for the 2010‐2011 season and in early‐mid March for the 2011‐2012 and 2015‐2016 seasons (Figure 1). In general, the peak week of influenza activity for each metric evaluated was the same as observed by CDC for the US nationally (Figure 2).^13^

Over all seasons analyzed, correlation between VA and CDC %ILI visits (*r* = .863), influenza hospitalizations (*r* = .953), positive influenza tests (*r* = .948), and percent of tests positive (*r* = .938) were strong. VA and CDC influenza laboratory data were most strongly correlated for the 2015‐2016 season (*r* = .998 for total positive tests and *r* = .994 for percent of tests positive), while hospitalizations (*r* = .985) and %ILI (*r* = .971) were most strongly correlated for the 2013‐2014 season (Table 2, *P* < .001 for each correlation).

**Table 2:** Correlation between VA and CDC FluView influenza indicators 2010‐2011 through 2015‐2016 seasons

Influenza Season	%Influenza‐like‐illness (ILI) visits*r* a	Influenza hospitalizations*r* a	Positive influenza tests*r* a	%Positive influenza tests*r* a
Combined	.863	.953	.948	.938
2010‐2011	.858	.928	.982	.981
2011‐2012	.824	.911	.978	.937
2012‐2013	.951	.969	.976	.980
2013‐2014	.971	.985	.989	.968
2014‐2015	.930	.954	.976	.972
2015‐2016	.899	.982	.998	.994

a *P*‐value < .001 for all correlations.

## 4 DISCUSSION

Our study highlights the burden of influenza in the largest integrated healthcare system in the United States. Of six seasons evaluated, the 2014‐2015 season demonstrated the highest activity with over 11 500 confirmed cases, 10 000 telephone triage encounters, 12 000 outpatient visits, and 4600 hospitalizations (hospitalization rate of 74.2 per 100 000 VA users). In contrast, the 2011‐2012 season had the lowest activity. Influenza A predominated in each season, which was consistent with national CDC FluView viral surveillance data.^11^ The wide range of metrics across influenza seasons demonstrates the yearly heterogeneity in severity of influenza disease, which is likely multifactorial. The high correlations with relatively small variation across season and metric type (0.824‐0.998, Table 2) are evidence of the robustness of the surveillance methods used by both VA and CDC.

Our surveillance data are routinely provided to a variety of stakeholders, including VA and CDC decision‐makers and could provide valuable information during another pandemic or vaccine shortage. Detailed facility‐level data are also regularly distributed to VA medical centers. VA Managerial Cost Accounting data were used by others to estimate the economic impact of influenza in VA for 2010‐2014. They estimated an annual cost of $1.2 billion attributable to influenza, including $6.2 million for ED visits, $36 million for hospitalizations, and $5.5 million for extended care rehabilitation and skilled nursing facility costs.^14^ These data are important to understand influenza epidemiology, local impacts, and effects on an entire healthcare system. It is also valuable for developing prevention strategies, containment measures and for resource allocation and supply distribution. For example, each VA facility makes independent decisions on how much vaccine to stock, and how much (if any) high‐dose vaccine to order, and develops criteria and strategies for distribution. Availability of high‐dose influenza vaccine formulation in VA increased during recent seasons. Epidemiologic and economic data are important drivers for these decisions.

This study has several limitations. Because not all persons with influenza sought medical care, and some sought treatment outside VA, we likely underestimated the true influenza burden in the VA patient population. Future analyses could account for influenza under‐detection by using a CDC multiplier method to adjust VA surveillance data.^3^ Administrative coding data were utilized for influenza hospitalizations, outpatient influenza and ILI visits, and telephone triage encounters. These data may not accurately capture true influenza burden due to coding inaccuracies and may not represent laboratory‐confirmed cases of influenza.^8^, ^9^, ^15^ Medical records and death certificates were not reviewed to determine whether influenza was a principal or contributing cause of death for those hospitalizations when listed as a diagnosis code. It is likely additional influenza‐related deaths occurred that were not captured by our surveillance. We are unable to report subtype data because it was not consistently performed for influenza A‐positive tests. Finally, influenza testing and vaccination data do not include tests or vaccinations ordered outside VA.

In conclusion, understanding influenza burden is valuable to VA leadership, facilities, and program offices for evaluating priorities and targeting resources for VA's annual influenza campaigns. Further enhancements to VA's public health surveillance systems may improve the accuracy of our influenza surveillance. Improving influenza vaccination rates is an important priority. Further expansion of VA's retail pharmacy vaccine program and mandatory influenza vaccination for employees are being considered by VA leadership. Additional research is needed to determine whether these or other prevention efforts will be effective in minimizing the clinical and economic burden of influenza in VA. Future plans for VA surveillance include merging vaccination data with influenza encounter and laboratory data to determine vaccination status among confirmed and probable cases and evaluating the effectiveness of high‐dose vs standard dose influenza vaccine in VA patients.
