# Supplementary material for: Epidemiology and burden of influenza in the U.S. Department of Veterans Affairs
Source: Influenza Other Respir Viruses. 2017 Dec 5;12(2):293–8. doi: 10.1111/irv.12512 (PMC5820422; doi:10.1111/irv.12512)
Supplement: Supplementary file 1 [file IRV-12-293-s001.docx]

**Supplementary Table 1**. **International Classification of Diseases, 9^th^ and 10^th^ Revisions, Clinical Modification (ICD-9-CM & ICD-10-CM) codes and code combinations included in the Veterans Affairs Influenza-like-illness (ILI) syndrome category.**

| **ICD-9-CM Code*** | **Description** |
| --- | --- |
| 079.89 | Other specified viral infection |
| 079.99 | Unspecified viral infection |
| 460. | Acute nasopharyngitis [common cold] |
| 462. | Acute pharyngitis |
| 465.8 | Acute upper respiratory infections of other multiple sites |
| 465.9 | Acute upper respiratory infections of unspecified site |
| 466.0 | Acute bronchitis |
| 480.9 | Viral pneumonia, unspecified |
| 484.8 | Pneumonia in other infectious diseases classified elsewhere |
| 485. | Bronchopneumonia, organism unspecified |
| 486. | Pneumonia, organism unspecified |
| 487.0 | Influenza with pneumonia |
| 487.1 | Influenza with other respiratory manifestations |
| 487.8 | Influenza with other manifestations |
| 488.01 | Influenza due to identified avian influenza virus with pneumonia |
| 488.02 | Influenza due to identified avian influenza virus with other respiratory manifestations |
| 488.09 | Influenza due to identified avian influenza virus with other manifestations |
| 488.11 | Influenza due to identified 2009 H1N1 influenza virus with pneumonia |
| 488.12 | Influenza due to identified 2009 H1N1 influenza virus with other respiratory manifestations |
| 488.19 | Influenza due to identified 2009 H1N1 influenza virus with other manifestations |
| 488.81 | Influenza due to identified novel influenza A virus with pneumonia |
| 488.82 | Influenza due to identified novel influenza A virus with pneumonia |
| 488.89 | Influenza due to identified novel influenza A virus with other respiratory manifestations |
| 490. | Bronchitis, not specified as acute or chronic |
| 780.60 + 784.1 | Fever, unspecified plus Throat pain |
| 780.60 + 786.2 | Fever, unspecified plus Cough |
| **ICD-10-CM Code*** | **Description** |
| B97.89 | Other viral agents as the cause of diseases classified elsewhere |
| J00 | Acute nasopharyngitis [common cold] |
| J02.9 | Acute pharyngitis, unspecified |
| J06.9 | Acute upper respiratory infection, unspecified |
| J09.X1 | Influenza due to identified novel influenza A virus with pneumonia |
| J09.X2 | Influenza due to identified novel influenza A virus with other respiratory manifestations |
| J09.X3 | Influenza due to identified novel influenza A virus with gastrointestinal manifestations |
| J09.X9 | Influenza due to identified novel influenza A virus with other manifestations |
| J10.00 | Influenza due to other identified influenza virus with unspecified type of pneumonia |
| J10.01 | Influenza due to other identified influenza virus with the same other identified influenza virus pneumonia |
| J10.08 | Influenza due to other identified influenza virus with other specified pneumonia |
| J10.1 | Influenza due to other identified influenza virus with other respiratory manifestations |
| J10.2 | Influenza due to other identified influenza virus with gastrointestinal manifestations |
| J10.81 | Influenza due to other identified influenza virus with encephalopathy |
| J10.82 | Influenza due to other identified influenza virus with myocarditis |
| J10.83 | Influenza due to other identified influenza virus with otitis media |
| J10.89 | Influenza due to other identified influenza virus with other manifestations |
| J11.00 | Influenza due to unidentified influenza virus with unspecified type of pneumonia |
| J11.08 | Influenza due to unidentified influenza virus with specified pneumonia |
| J11.1 | Influenza due to unidentified influenza virus with other respiratory manifestations |
| J11.2 | Influenza due to unidentified influenza virus with gastrointestinal manifestations |
| J11.81 | Influenza due to unidentified influenza virus with encephalopathy |
| J11.82 | Influenza due to unidentified influenza virus with myocarditis |
| J11.83 | Influenza due to unidentified influenza virus with otitis media |
| J11.89 | Influenza due to unidentified influenza virus with other manifestations |
| J12.9 | Viral pneumonia, unspecified |
| J18.8 | Other pneumonia, unspecified organism |
| J18.9 | Pneumonia, unspecified organism |
| J20.9 | Acute bronchitis, unspecified |
| J40 | Bronchitis, not specified as acute or chronic |
| R50.9 + R05 | Fever, unspecified + Cough |
| R50.9 + R07.0 | Fever, unspecified + Pain in throat |

*ICD-9 codes were utilized prior to October 1, 2015 and ICD-10 codes were utilized beginning on October 1, 2015.
